# The Battle of the Equations: A Systematic Review of Jump Height Calculations Using Force Platforms

**DOI:** 10.1007/s40279-024-02098-x

**Published:** 2024-10-19

**Authors:** Ingrid Eythorsdottir, Øyvind Gløersen, Hannah Rice, Amelie Werkhausen, Gertjan Ettema, Fredrik Mentzoni, Paul Solberg, Kolbjørn Lindberg, Gøran Paulsen

**Affiliations:** 1 Department of Physical Performance, Norwegian School of Sport Sciences, Oslo, Norway; 2 Norwegian Olympic and Paralympic Committee and Confederation of Sports, Oslo, Norway; 3 Smart Sensors and Microsystems, SINTEF Digital, Oslo, Norway; 4 Intelligent Health Initiative, Section for Pharmacy, Department of Life Sciences and Health, Oslo Metropolitan University, Oslo, Norway; 5 Department of Neuromedicine and Movement Science, Center for Elite Sports Research, Norwegian University of Science and Technology, Trondheim, Norway; 6 Department of Sport Science and Physical Education, Faculty of Health and Sport Sciences, University of Agder, Kristiansand, Norway

## Abstract

Vertical jump height measures our ability to oppose gravity and lower body neuromuscular function in athletes and various clinical populations. Vertical jump tests are principally simple, time-efficient, and extensively used for assessing athletes and generally in sport science research. Using the force platform for jump height estimates is increasingly popular owing to technological advancements and its relative ease of use in diverse settings. However, ground reaction force data can be analyzed in multiple ways to estimate jump height, leading to distinct outcome values from the same jump. In the literature, four equations have been commonly described for estimating jump height using the force platform, where jump height can vary by up to $\sim$ 15 cm when these equations are used on the same jump. There are advantages and disadvantages to each of the equations according to the intended use. Considerations of (i) the jump type, (ii) the reason for testing, and (iii) the definition of jump height should ideally determine which equation to apply. The different jump height equations can lead to confusion and inappropriate comparisons of jump heights. Considering the popularity of reporting jump height results, both in the literature and in practice, there is a significant need to understand how the different mathematical approaches influence jump height. This review aims to investigate how different equations affect the assessment of jump height using force platforms across various jump types, such as countermovement jumps, squat jumps, drop jumps, and loaded jumps.

## Key Points

**Table:** 

Using a force platform, jump height estimations can vary by ~15 cm depending on the equations applied. Four equations are commonly used: two define jump height from take-off, and two from standing.
Jump height estimated from take-off with the take-off velocity (ToV) equation suits both within- and between-athlete comparisons during unloaded jumps. Deriving jump height from take-off using the flight time (FT) equation is suitable for within-athlete comparisons and recommended for loaded jumps but requires athletes to land with extended legs for accurate measurements.
Deriving jump height from standing using the take-off velocity plus displacement (ToV+D) or the displacement (DIS) equation is more representative of an athletic setting, but errors may arise due to integration drift. These approaches are less suitable for squat jumps. If implementing backward integration, athletes must remain still after landing.
Jump height measurements should not be compared between studies or testing facilities without considering the context of the methodology applied.

## Background

Maximal vertical jump height is a common metric used to evaluate the neuromuscular capacity of the lower extremities, and the vertical jump test is the generic test most frequently used for this purpose [16, 22, 39, 44, 52, 55, 71, 72, 87, 102, 104]. The height we can jump from the ground indicates our ability to oppose gravity [11, 89], which explains why the maximal vertical jump is one of the most popular tests for assessing general motor ability and movement performance [1, 5, 12, 15, 19, 21, 44, 45, 71, 76, 87, 89, 93]. However, to exemplify, in the context of soccer, mean jump heights between similar populations have been reported to vary by $\sim$ 17 cm [40, 101], which far exceeds what could be attributed to variances in movement performance within the same sport. It is important to note that these discrepancies may arise from methodological differences in the studies conducted. Indeed, obtaining a correct measure of jump height is not a straightforward task [102].

**Fig. 1:**
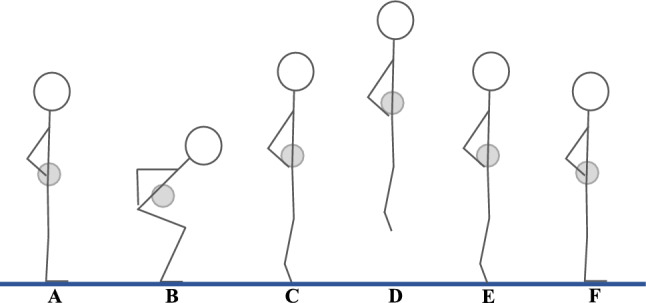
A sketch of a countermovement jump (CMJ). **A** Initial standing still prior to the CMJ. **B** Countermovement (unloading/braking). **C** Take-off instant. **D** Apex of the flight phase. **E** Landing instant. **F** Landed after the CMJ. See phase definitions in Refs. [36, 63]. Gray-filled circles represent a graphical estimation of the center of mass

According to the laws of Newtonian physics, vertical jump height is determined by the elevation of the center of mass (CoM). Jump height can, therefore, be estimated by tracking the displacement of the CoM throughout a jump, which is exemplified as a countermovement jump (CMJ) in Fig. 1 [24, 61, 71, 87].

To date, three-dimensional (3D) motion capture systems provide the most direct estimates of CoM kinematics [24, 71, 87]. However, the equipment needed for these measures poses significant environmental constraints, especially for in-field testing of athletes—because they are time-consuming and require expensive equipment and analytical expertise [73, 92, 104]. For these reasons, other, simpler methods to calculate jump height have been developed, such as force platforms [60, 63], contact mats [14], photoelectric cells [32], linear position transducers [21], and smartphone (video/accelerometer) applications [5, 28]. These methods have been developed in addition to even simpler measurements, such as the Sargent test [55, 74, 89]. In their systematic review, Xu et al. [102] concluded that, of all the equipment available for jump height calculations, the force platform is the most appropriate for estimating jump height, as it eliminates some of the errors associated with both the direct and indirect methods listed above. Indeed, the force platform is increasingly popular for jump assessments as it is the only technology that allows direct kinetic analysis of a jump [8, 56, 60, 61, 63]. Moreover, force platform systems are steadily becoming more accessible for testing athletes. They are no longer restricted to the laboratory but exist in portable and more affordable versions, including software that provides instant results. In fact, these trends are mirrored in the literature by a growing number of publications related to the implementation of the force platform for jump assessments in field settings [6, 9, 17, 29, 30, 34, 35, 48, 53, 63–66, 82, 85, 100, 102].

**Fig. 2:**
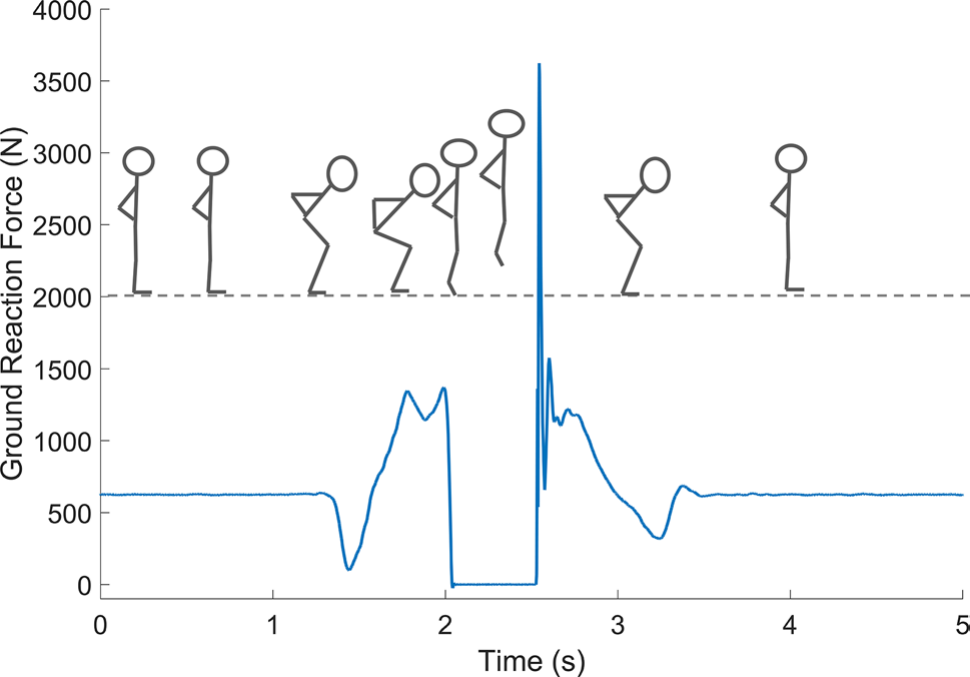
The vertical ground reaction forces (N) measured by a force platform (not depicted) are plotted as the blue solid line against time (s) during a countermovement jump (CMJ). The stick figures represent the time-synchronized movement of a person during the CMJ on the ground (depicted as the dashed gray line)

A force plate is a device that typically measures the 3D ground reaction forces (GRFs), with the vertical component being the most commonly applied in jump-related research (Fig. 2) [60]. The laws of mechanics allow the vertical GRFs to be used to calculate jump height, but different mathematical approaches can be applied [56, 63, 102, 104].

### Governing Equations

The fundamental understanding of a jump lies in Newton’s second law of motion,

1 $$\begin{aligned} \Sigma { \varvec{F} } = m \varvec{a}, \end{aligned}$$

which states that the sum of forces acting on an object’s CoM, $\Sigma \varvec{F}$, is equal to its mass, *m*, multiplied by its acceleration, $\varvec{a}$. Bold font is here used to indicate a vector quantity. The net acceleration of the CoM from Eq. (1) is

2 $$\begin{aligned} \varvec{a} = \frac{\!d\varvec{v}}{\!dt}, \end{aligned}$$

with $\varvec{v}$ from Eq. (2) being the velocity of the CoM,

3 $$\begin{aligned} \varvec{v} = \frac{\!d\varvec{s}}{\!dt}, \end{aligned}$$

where *t* is time, and $\varvec{s}$ is the displacement of the CoM. Combining Eqs. (1) and (2), and integrating from the initial, *i*, to the final, *f*, state with respect to the movement of interest, yields the impulse–momentum theorem,

4 $$\begin{aligned} \int _{t_i}^{t_f} \Sigma \varvec{F} dt = m \varvec{v}_{f} - m \varvec{v}_{i}. \end{aligned}$$

The impulse–momentum theorem describes how the accumulation of force over time (impulse) creates changes in momentum (mass multiplied by velocity), which is fundamental for athletic movements such as vertical jumping. The following sections explain the rationale for applying the impulse–momentum theorem for jump height calculations.

The mechanical energy during a jump is the combination of potential and kinetic energy. Lifting the CoM a vertical distance implies an increase in the potential energy. Potential energy represents the energy due to the position of the CoM in a gravitational field, and the value at the start of the jump is typically chosen as the reference (zero) point for potential energy. Note that the reference point for potential energy can be defined arbitrarily and may vary depending on the context and analyses being performed. In jumping, the reference point for potential energy is often either the participant’s CoM position while standing still or at the take-off position, i.e., the heel-rise makes the difference (Fig. 1). Jump height can thus be defined relative to standing ($\hbox {JH}_{\text {standing}}$) or relative to take-off ($\hbox {JH}_{\text {takeoff}}$), depending on the reference point applied.

The velocity of the CoM determines the kinetic energy. Since mechanical energy is assumed to be conserved throughout the flight phase, the sum of the potential and kinetic energy is constant,

5 $$\begin{aligned} mgz + \frac{1}{2} m w^2 = \text {constant}, \end{aligned}$$

where *z* is the vertical coordinate of the CoM, *w* is the vertical velocity component of the CoM, and *g* is the gravitational acceleration.

### Jump Height from Take-Off Velocity

$\hbox {JH}_{\text {takeoff}}$ can be estimated from the take-off velocity by assessment of Eq. (5). During a jump, the CoM moves upward against gravity. After take-off, gravity gradually slows the upward movement until the CoM reaches its highest point of the jump (the apex). At the apex, the vertical kinetic energy of the CoM is zero. This is because the CoM has momentarily stopped moving upwards, and the gravitational potential energy is at its maximum since the CoM has reached its highest position with respect to the reference point. As the CoM descends, potential energy is converted back to kinetic energy. In other words, the mechanical energy remains constant throughout the jump (Eq. (5)). So, to jump as high as possible, the aim will be to increase the velocity of the CoM at take-off. Defining the take-off as point C, the apex as point D (see definitions in Fig. 1), and the difference in the vertical displacement of the CoM between points C and D as *h*, Eq. (5) simplifies to

6 $$\begin{aligned} w_C^2 = w_D^2 + 2 g(z_D-z_C). \end{aligned}$$

Defining the jump height as $h = z_D-z_C$, and solving for $\hbox {JH}_{\text {takeoff}}$, yields

7 $$\begin{aligned} {h = \frac{w_C^2}{2g}}. \end{aligned}$$

**Fig. 3:**
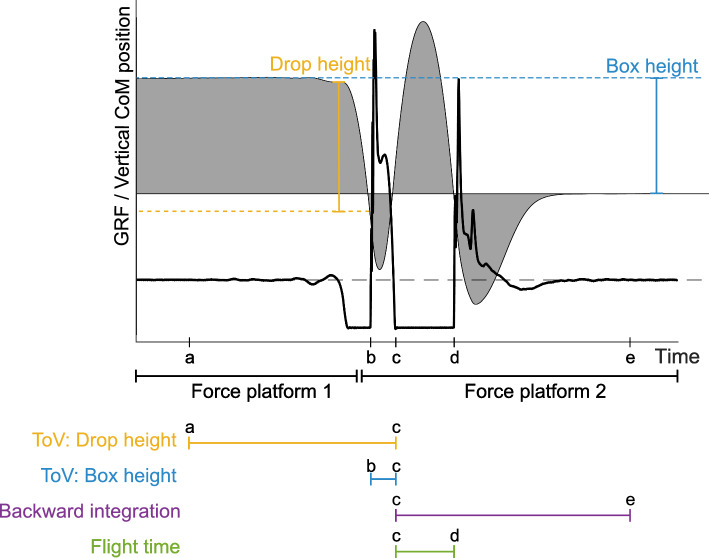
Ground reaction forces (GRFs, solid black line) and vertical center of mass (CoM) trajectory (shaded gray area, displacement from standing still on the ground) plotted against time during a drop jump. Force platform 1 measures GRFs through the box, while force platform 2 measures GRFs during the jump and landing. The dashed black line indicates body weight. The dashed blue line illustrates the height of the box from which the person drops (i.e., the “box height”). The distance between the dashed blue and yellow lines indicates the drop in CoM height before contacting force platform 2 (i.e., the “drop height”). The yellow line segment below the axis represents GRFs from standing still to take-off, used with the “ToV: drop height” method. The blue line segment represents GRFs between the time the person lands on the force platform (after the drop) and take-off, which is used together with box height in the “ToV: box height” method. The purple line segment represents GRFs between take-off and standing still after landing, used with the “backward integration” method. The green line segment represents the time in the air when no force is measured by the force platform, used by the “flight time” method

For the purpose of this review, Eq. (7) will be referred to as the take-off velocity method (ToV). To estimate $\hbox {JH}_{\text {takeoff}}$ from Eq. (7), $w_C$ must be determined from Eq. (4). For convenience, the force is integrated from a state where velocity is zero (e.g., during quiet standing), thus eliminating the last term of Eq. (4). The assumption of zero initial velocity is presumably satisfied when performing the CMJ or the squat jump (SJ) because the participant starts from a quiet standing (CMJ) or squat (SJ) position. When performing a drop jump (DJ), the initial velocity is not zero, as the person drops off a box prior to contact with the force plate. If the drop height is known, the initial velocity can be estimated using Eq. (5). One way of estimating drop height is to simply use box height as a measure of drop height (dashed blue line in Fig. 3) [4, 7, 9, 47, 50, 57, 65, 77]. However, owing to changes in posture (and CoM) during descent, the drop height is not easily approximated by the height of the box [12, 50, 65]. If the box from which the person drops is located on a second force platform (force platform 1 in Fig. 3), the touchdown velocity can be determined analogously to the take-off velocity by using the impulse–momentum theorem (Eq. (5)) [4, 46, 65].

The double force platform method is usually set as a criterion for DJ analyses because obtaining GRF–time data of the jumper prior to the drop (yellow line in Fig. 3) allows for a more accurate estimate of touchdown velocity (Eq. (4)), compared with estimating touchdown velocity on the basis of the box height. With the double force platform procedure, the actual displacement of the CoM as the jumper steps off the box is obtained by double integrating the force data from the force platform from where the person is standing prior to the jump (where the box is located). The displacement of the CoM as the jumper steps off the box is then used to obtain the initial velocity condition through Eq. (5) [4, 12, 20, 23, 65]. Such an approach is, however, practically less feasible as it requires practitioners (and researchers) to possess two force platforms [65]. To circumvent this issue, some researchers have reported DJ heights to be analyzed with the initial velocity of zero taken from the end of the jump (at a period after the landing), and from this, simply applying Eq. (7), integrating backward to take-off [4, 46, 65, 99]. Obtaining a velocity of zero at the end of the jump requires the participants to stand still after they have landed. Alternatively, the flight-time approach can be used (see Sect. 1.4) [4].

### Jump Height from Standing

When the reference for potential energy is the standing position, the same principles as discussed above are addressed, although the velocity of the CoM needs to be integrated to obtain the displacement of the CoM (Eqs. (2) and (3)). In this context, $\hbox {JH}_{\text {standing}}$ can be estimated as the maximum displacement of the CoM during the jump (from standing still to the apex of the jump; Fig. 1 from point A to point D),

8 $$\begin{aligned} {h = \textrm{max} \left( \int _A^E \int _A^E a_z \ dt \ dt \right) }, \end{aligned}$$

or by finding the displacement of the CoM at take-off and combining it with Eq. (7),

9 $$\begin{aligned} {h = \int _A^C \int _A^C a_z \ dt \ dt + \frac{w_C^2}{2g}}. \end{aligned}$$

For the purpose of this review, Eq. (8) will be referred to as the displacement method (DIS) and Eq. (9) will be referred to as the take-off velocity plus displacement method (ToV+D). Note that Eqs. (8) and (9) can also be calculated by performing a backward integration, starting from the end of the jump. Practically, this means that, for Eq. (8), integration is from point F to point A in Fig. 1, while for Eq. (9), the integration is from point F to point E in Fig. 1. For the latter, jump height is then defined as $\hbox {JH}_{\text {landing}}$, instead of $\hbox {JH}_{\text {takeoff}}$. It is important to mention that both backward and forward integration can only be performed over a period where all force information and a state condition (i.e., speed and position) at one point are known.

The push-off distance in vertical jumping, which is included in Eqs. (8) and (9), could have performance implications, as the push-off phase is a critical aspect of the jump technique that can significantly affect the height achieved [10–12, 27, 75, 95, 96]. Whether jump height is best determined when defined with or without the heel-rise requires further discussion from a practical perspective and will be elucidated later.

### Flight Time Estimation

An alternative to estimating jump height from the vertical GRFs and gravity is to measure flight time. If considering the jumping body as an object under constant acceleration (the gravitational acceleration) during the flight phase of the jump, Eq. (2) implies a vertical velocity component linear in time,

10 $$\begin{aligned} w = w_0 - g t. \end{aligned}$$

The constant $w_0$ can be determined from the fact that, at the apex, at time $t_a$, the vertical velocity is momentarily zero. Consequently, $w_0 = g t_a$. Further, it follows from Eq. (3) that the vertical displacement is parabolic in time

11 $$\begin{aligned} z = z_0 + g t_a t - \frac{1}{2} g t^2. \end{aligned}$$

At $t = t_a$, the difference in displacement is equal to the jump height, $z - z_0 = h$. Consequently,

12 $$\begin{aligned} h = \frac{1}{2} g t_a^2. \end{aligned}$$

Since the jump follows a parabolic path due to gravity, as evident in Eq. (11), the time to reach the apex of the jump is half the total flight time,

13 $$\begin{aligned} t_a = \frac{t_\textrm{flight}}{2}. \end{aligned}$$

Thus, the relation between $\hbox {JH}_{\text {takeoff}}$ and the total flight time is

14 $$\begin{aligned} {h = \frac{g t_\textrm{flight}^2}{8}}. \end{aligned}$$

For the purpose of this review, Eq. (14) will be referred to as the flight-time method (FT). Even though Eq. (14) can be, and is, used to calculate $\hbox {JH}_{\text {takeoff}}$ from force plate recordings [1, 3, 18, 69, 71, 79, 99], it is used more frequently with technologies such as contact mats and smartphone applications, as this method does not directly rely on the GRF–time tracings, but simply the detection of take-off and landing [14, 71, 87, 91, 102, 104].

### The Four Different Jump Height Equations

The four equations presented above each have their specific inputs that are required to estimate jump height from the GRF–time tracings. How well these inputs can be satisfied depends on the jump modality tested. The CMJ, SJ, DJ, and loaded jumps are examples of jump modalities widely used in a sports context and have distinct movement characteristics—which will affect how accurately each of the above-mentioned equations estimates jump height.

An additional factor to consider is the different jump height definitions presented in the equations above—where ToV and FT estimate jump height relative to take-off, while ToV+D and DIS estimate jump height relative to standing. The heel-rise is the most obvious difference between defining jump height relative to take-off or standing. The heel-rise is relatively consistent for each individual over time [97]. Thus, if comparing jump height results within an athlete over time, defining jump height relative to take-off is a simpler and faster approach that is also less prone to errors (drift) compared with the ToV+D and DIS methods.

However, estimating jump height relative to take-off does not account for the work and power required to raise the CoM from rest. During standing (point A in Fig. 1) and at the apex of the jump (point D in Fig. 1), the body is at rest. The lower extremity muscles will produce negative or positive work between these positions. Thus, power will be underestimated when jump height has been defined relative to take-off, if power has been estimated from jump height alone. Also, while foot length is consistent within an individual over time, it will differ between people. Different foot lengths imply a difference in the work and power needed to lift the CoM from standing to toe-off. In addition, the plantar flexion angle at take-off can influence the heel-rise. Therefore, comparing jump height between athletes using ToV or FT does not account for the differences in heel-rise and, therefore, the increased work and power needed to lift the CoM from standing to take-off.

### Summary

The scientific literature conveys that the impulse–momentum theorem (Eqs. (4), (8), (9)) provides better validity and reliability of jump height estimations, compared with the flight-time approach (Eq. (14)) [4, 13, 18, 26, 33, 40, 41, 48, 49, 51, 54–56, 58, 62, 69, 78, 82, 84, 85, 94, 98, 99, 102, 103]. Nevertheless, both the impulse–momentum equations and the flight-time approach are used in practice with force platforms. Indeed, it remains unexplored how the different jump height equations, reported in the literature and used in practice, are best suited for jump height calculations for several of the common jump modalities, such as the CMJ, SJ, DJ, and loaded jumps.

Researchers have attempted to provide some guidelines regarding which equation is best suited for jump height calculations using force platforms. From these studies, differences in jump height estimations in the range of 0.6–15 cm have been reported when applying different equations on the same jump [1, 4, 18, 42, 50, 67, 69, 79, 80, 86, 97, 99, 103], highlighting how the equations presented above cannot be used interchangeably. Still, there seems to be an inconsistency between the equations recommended by researchers and the equations most frequently used, both in research and in practice. The observed discrepancies may stem from guidelines that are either too vague, suggesting widely different approaches, or neglecting the practical context, which makes it challenging to determine the appropriate jump height equation for different settings using the force platform. We understand that, in real-world scenarios, choosing the preferred equations can be challenging or even impossible owing to the use of proprietary software. However, we aim to educate practitioners so that they consider this factor when procuring new equipment.

The aim of this review is to explore how the choice of different equations affects the estimated jump height using force platforms to assess CMJ, SJ, DJ, and loaded jumps. While we acknowledge that GRF signal processing also influences jump height estimates using force platforms [8, 25, 37, 49, 68, 81, 83, 86, 88, 90, 94, 98], this review solely addresses the equations used to calculate jump height. By examining the expected differences in jump height and the underlying reasons that arise from using distinct mathematical approaches for force-platform-based jump height assessments, this review provides insights that can inform practitioners and researchers on optimizing their approach to jump height evaluations using the force platform.

## Methods

A literature search was conducted between April 2021 and May 2024 in PubMed, SPORTDiscus, and Web of Science.

In PubMed, the following search was conducted: ("vertical jump*" OR "squat jump*" OR "countermovement jump*" OR "drop jump*" OR "jump height*") AND ("force plate*" OR "force platform*" OR "biomechanical phenomena"[mesh] OR "ground reaction force*") AND (velocity OR filter* OR "body weight"[mesh] OR "flight time*" OR movement/physiology[mesh] OR motion[mesh] OR exercise test/methods[mesh] OR movement/physiology[mesh]) NOT (phone OR app OR device* OR photocell* OR ACL OR inertial*).

In Web of Science and SPORTDiscus, the following search was conducted: "vertical jump*" OR "squat jump*" OR "countermovement jump*" OR "drop jump*" OR "jump height*"AND "force plate*" OR "force platform*" OR “biomechanical phenomena” OR "ground reaction force*" AND velocity OR filter* OR "body weight" OR "flight time*" OR movement OR motion OR "exercise test*" NOT phone OR app OR device* OR photocell* OR ACL OR inertial*.

The inclusion criteria were: (i) using the force platform for jump height calculations; (ii) comparing at least two of the four included jump height equations: Eqs. (7), (8), (9), or (14); (iii) the equation used to calculate jump height was described; (iv) comparisons between the equations were based on force platform recordings (but the criterion could be obtained from 3D motion capture); (v) the participants were healthy and had no reported disabilities; (vi) the papers were peer-reviewed; (vii) the papers were original empirical studies, and (viii) the papers were written in English. Studies where equipment such as contact mats were placed on top of the force platform were excluded.

## Results

### Literature Search

The search from the three databases retrieved a total of 2528 results. After removing duplicates between databases, 1568 peer-reviewed articles were obtained. First, papers were screened by title, excluding papers that clearly addressed issues such as training interventions, muscle activity, coordination patterns, attentional focus on jump height outcomes, etc. After these exclusions, 82 papers were included for further analyses.

**Fig. 4:**
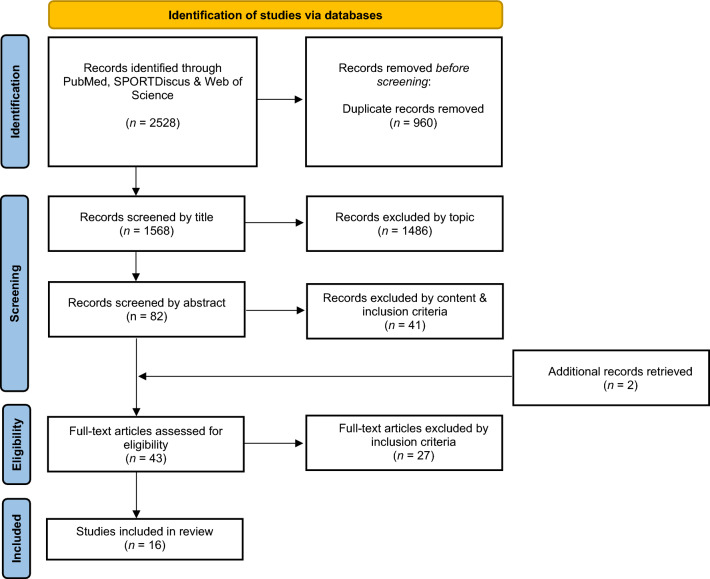
Literature search flowchart

The abstracts were carefully read, and 41 articles were excluded at this stage as they did not provide any direct comparisons in jump height when calculated with different equations using force platforms. The remaining 41 papers were thoroughly read. Two additional papers were retrieved from the reference lists at this stage. Of the remaining 43 papers, 16 papers addressed different equations for jump height calculations using the force platform and were included in the present review (Fig. 4).

### Quality Grading

The quality grading system used in this study was adapted and modified from Xu et al. [102] for the purpose of this review. Each paper was graded using five criteria on a scale from 0 to 2 (0 = no, 1 = maybe/unclear, 2 = yes). Thus, each paper’s maximum score was 10 (Table 1). We considered 0−3 points as low quality, 3–6 points as moderate quality, and 7–10 as high quality. Only papers with scores of 7–10 were included in this review (Table 2).

**Table 1:** Quality grading system

Criteria no.	Item	Score
1	Participants described appropriately (age, sex, height, weight, training)	0–2
2	Procedures described (equations, equipment, jump modality)	0–2
3	Results detailed (mean, standard deviation, or absolute jump height)	0–2
4	Appropriate statistics (reliability and/or significant differences)	0–2
5	Conclusion insightful (clear, practical application, future directions)	0–2
Total		0–10

**Table 2:** Results of quality grading scoring

References	Criteria no.					
	1	2	3	4	5	Total
Kibele [49]	2	2	2	2	1	9
Baca [4]	2	2	1	1	2	8
Aragón-Vargas [1]	2	2	2	2	2	10
Moir [69]	2	2	2	1	2	9
Pérez-Castilla et al. [80]	2	2	2	2	2	10
Heishman et al. [42]	2	2	1	1	2	8
Pérez-Castilla and García-Ramos [79]	2	2	2	2	2	10
Wank and Coenning [99]	2	1	1	2	1	7
Chiu and Dæhlin [18]	2	1	2	1	1	7
Wade et al. [97]	2	2	2	2	2	10
Yamashita et al. [103]	2	2	2	2	2	10
Jørgensen et al. [46]	2	2	2	2	1	9
McMahon et al. [65]	2	2	2	1	2	9
Merrigan et al. [67]	2	2	2	2	2	10
Pinto and Callaghan [86]	2	2	2	2	2	10
Wade et al. [98]	2	2	1	2	2	9

### Grouping Equations

Most of the included studies applied different names for the same equation. Thus, for the purpose of this review, these equations have been divided into groups to make the comparisons more understandable. These groups are as follows:

FT: flight-time (Eq. (14))

ToV: take-off velocity (Eq. (7))

ToV+D: take-off velocity + the displacement of the CoM until take-off (Eq. (9))

DIS: maximum displacement of the CoM via double integration of the vertical GRFs (Eq. (8))

### Study Characteristics

Of the 16 papers included in this review which addressed whether the flight-time (Eq. (14)) or various impulse–momentum equations (Eqs. (7) to (9)) should be applied to calculate jump height, 10 papers analyzed unloaded CMJs, 1 analyzed loaded CMJs, 1 analyzed unloaded SJs, 1 analyzed loaded SJs and 2 papers analyzed DJs. Two additional papers analyzed DJ heights using single versus two force platforms. Of the 16 papers, 1 addressed differences in jump height performed with or without arm swings. For the remaining papers, jumps were performed with arms on the hip (Table 3).

### Agreement between Equations

FT has been observed to overestimate $\hbox {JH}_{\text {takeoff}}$ compared with ToV by on average 2–6%, for unloaded CMJs, SJs, and DJs. For loaded jumps, FT has been observed to underestimate $\hbox {JH}_{\text {takeoff}}$ by 1–21%, for CMJs and SJs. Compared with ToV+D and DIS, FT and ToV have been observed to underestimate $\hbox {JH}_{\text {standing}}$ by on average 26–45%, for unloaded CMJs and SJs (Table 3).

For DJs, the single force platform methods have been observed to over- and underestimate jump height by 4% and 3%, respectively (Table 3).

**Table 3:** Absolute differences in jump height between equations using force platforms, and the corresponding authors’ recommendations on which equation to use

Study	Jump types	Equations	Jump height (cm)		Recommendations
Kibele [49]	CMJ	FT	30.8 ± 7.5		FT $=$ ToV
		ToV	30.2 ± 7.2		
Aragón-Vargas [1]	CMJ	FT	40.2 ± 6.7		FT
		ToV	36.1 ± 6.6		
Moir [69]	CMJ		*Men*	*Women*	ToV
		FT	35.8 ± 5.9	21.6 ± 4.6	
		ToV	34.8 ± 5.6	20.7 ± 4.6	
		ToV$+$D	46.7 ± 5.6	30.7 ± 4.7	
Heishman et al.$^{\textrm{a}}$ [42]	CMJ		*With arms*	*Without arms*	With arms: FT; Without arms: FT $=$ ToV
		FT	43.9 ± 0.3	36.4 ± 0.6	
		ToV	44.5 ± 2.4	35.8 ± 0.6	
Yamashita et al. [103]	CMJ	FT	42.1 ± 8.1	42.1 ± 8.1	ToV
		ToV	39.6 ± 7.4	39.6 ± 7.4	
Merrigan et al. [67]	CMJ	FT	27.3 ± 6.4	27.3 ± 6.4	No clear recommendations
		ToV	26.0 ± 6.4	26.0 ± 6.4	
Pérez-Castilla et al.$^{\textrm{b}}$ [79]	Loaded CMJs	FT	*Free weights*	*Smith machine*	Free weights: FT $=$ ToV; Smith machine: FT
		*17 kg*	25.3 ± 3.4	26.0 ± 3.5	
		*30 kg*	20.7 ± 2.8	21.1 ± 3.5	
		*45 kg*	15.9 ± 2.6	16.1 ± 3.0	
		*60 kg*	10.9 ± 2.3	11.1 ± 2.9	
		*75 kg*	8.6 ± 2.5	9.0 ± 2.6	
		ToV	*Free weights*	*Smith machine*	
		*17 kg*	24.9 ± 3.1	25.5 ± 3.4	
		*30 kg*	20.8 ± 2.7	22.0 ± 3.2	
		*45 kg*	16.0 ± 2.2	16.5 ± 3.0	
		*60 kg*	10.9 ± 2.0	11.0 ± 2.7	
		*75 kg*	9.0 ± 2.2	9.6 ± 2.8	
Pérez-Castilla et al.$^{\textrm{b}}$ [80]	Loaded SJs	FT	*Free weights*	*Smith machine*	Free weights: FT $=$ ToV; Smith machine: FT
		*17 kg*	21.8 ± 3.7	21.4 ± 3.8	
		*30 kg*	17.4 ± 3.5	17.3 ± 3.2	
		*45 kg*	13.4 ± 2.5	13.0 ± 3.2	
		*60 kg*	9.0 ± 2.6	8.6 ± 2.8	
		*75 kg*	7.2 ± 2.6	6.6 ± 2.6	
		ToV	*Free weights*	*Smith machine*	Free weights: FT $=$ ToV; Smith machine: FT
		*17 kg*	21.9 ± 3.9	22.7 ± 3.8	
		*30 kg*	18.0 ± 3.4	19.6 ± 3.3	
		*45 kg*	13.9 ± 3.2	16.2 ± 3.6	
		*60 kg*	9.5 ± 3.3	12.4 ± 3.2	
		*75 kg*	8.1 ± 2.9	10.7 ± 3.1	
Wade et al. [97]	SJ	FT			Equations not using the force platform
	CMJ	*CMJ*	32.3 ± 1.3		
		ToV			
		*CMJ*	31.3 ± 1.1		
		*SJ*	28.1 ± 1.4		
		ToV$+$D			
		*CMJ*	43.2 ± 1.5		
		*SJ*	27.0 ± 7.4		
Pinto and Callaghan [86]	CMJ	ToV	29.6 ± 6.1		ToV$+$D
		ToV$+$D	41.7 ± 6.0		
Wank and Coenning$^{\textrm{c}}$ [99]	CMJ	FT			DIS
	SJ	*CMJ*	43.8 ± 6.1		
	DJ	*SJ*	38.5 ± 6.1		
		*DJ*	38.1 ± 5.7		
		ToV			
		*CMJ*	41.2 ± 5.5		
		*SJ*	36.4 ± 5.4		
		*DJ*	35.9 ± 4.2		
		DIS			
		*CMJ*	57.8 ± 6.6		
		*SJ*	50.9 ± 7.5		
		*DJ*	49.6 ± 5.7		
Chiu and Dæhlin [18]	CMJ	FT	30.9 ± 9.4		ToV$+$D
		ToV	29.8 ± 8.9		
		ToV$+$D	42.0 ± 9.4		
		DIS	43.2 ± 1.5		
Wade et al. [98]	CMJ		*Submaximal*	*Maximal*	
		ToV$+$D	30.4 ± 5.0	38.7 ± 6.3	ToV$+$D$_{\textrm{b}} =$ ToV$+$D
		ToV$+$D$_{\textrm{b}}$	30.5 ± 5.2	38.9 ± 6.3	
		FT	20.7 ± 5.3	28.7 ± 6.8	
Baca$^{\textrm{c}}$ [4]	DJ	FT	33.9 ± 6.8	33.9 ± 6.8	ToV$_{\textrm{2fp}}$
		ToV$_{\textrm{2fp}}$	32.6 ± 2.3	32.6 ± 2.3	
		ToV$_{\textrm{b}}$	32.6 ± 4.3	32.6 ± 4.3	
		ToV$_{\textrm{td}}$	33.9 ± 13.9	33.9 ± 13.9	
Jørgensen et al. [46]	DJ	ToV$_{\textrm{2fp}}$	33.0 ± 6.0	33.0 ± 6.0	ToV$_{\textrm{2fp}}$
		ToV$_{\textrm{b}}$	32.0 ± 6.0	32.0 ± 6.0	
McMahon et al. [65]	DJ	ToV$_{\textrm{2fp}}$	28.0 ± 6.0	28.0 ± 6.0	ToV$_{\textrm{pm}} =$ ToV$_{\textrm{2fp}}$
		ToV$_{\textrm{pm}}$	28.0 ± 6.0	28.0 ± 6.0	

*CMJ* countermovement jump, *SJ* squat jump, *DJ* drop jump, *FT* flight-time (Eq. (13)), *ToV* take-off velocity (Eq. (6)), *ToV+D* take-off velocity $+$ displacement of the center of mass (CoM) at take-off (Eq. (8)), *DIS* maximum displacement of the CoM (Eq. (7)). For DJs: *ToV*$_{\textrm{2fp}}$ Eq. 6 applying the double force platform ($_{\textrm{2fp}})$ method, *ToV*$_{\textrm{b}}$ Eq. 6 applying a backward integration ($_{\textrm{b}})$ approach, *ToV*$_{\textrm{pm}}$ Eq. 6 applying a proposed method ($_{\textrm{pm}})$, *ToV*$_{\textrm{td}}$ Eq. 6 applying the touchdown ($_{\textrm{td}})$ velocity as the initial velocity. Please see Sect. 4.2 for further details on the DJ equations. In Wade et al., ToV $+$ D is their forward integration method, while ToV $+$ D$_{\textrm{b }}$ represents their backward integration procedure, where $_{\textrm{b}}$ denotes backward

$^{\textrm{a}}$The standard deviation (SD) was calculated on the basis of the typical error (TE) presented by the authors by rearranging the equation they presented to calculate the TE ($\frac{\textrm{SD}}{\sqrt{2} })$

$^{\textrm{b}}$Only data from the second session are presented

$^{\textrm{c}}$The results were extracted using WebPlotDigitizer, version 4.6.0. The absolute values presented in Ref. [4] were calculated from the percentage difference from the reference value

### Reliability between Equations

On average across all papers, FT demonstrated better within- and between-session reliability compared with ToV and ToV+D (Table 4).

**Table 4:** Reliability between jump height equations using force platforms

Study	Participants	Jump type	Type of reliability	Number of jumps	Reliability measures	Equations	Results
Aragón-Vargas [1]	52 physically active college students	CMJ	Within-session	5	SEM	FT	1.2 cm
Aragón-Vargas [1]	52 physically active college students	CMJ	Within-session	5	SEM	ToV	1.8 cm
Yamashita et al. [103]	3 females & 24 males	CMJ^a^	Within-session	3	ICC_1,1_	FT	0.96
Yamashita et al. [103]	3 females & 24 males	CMJ^a^	Within-session	3	ICC_1,1_	ToV	0.98
Merrigan et al. [67]	87 female & 25 male NCAA athletes	CMJ	Within-session	2	CV, ICC, SEM	FT	CV = 2.9%, ICC = 0.97, SEM = 1.03 cm
Merrigan et al. [67]	87 female & 25 male NCAA athletes	CMJ	Within-session	2	CV, ICC, SEM	ToV	CV = 3.2%, ICC = 0.87, SEM = 2.44 cm
Heishman et al. [42]	8 female & 14 male Division 1 NCAA collegiate basketball players	CMJ (without arm swing)	Within- & between-sessions	3	CV, ICC, SWC	FT	Within; CV = 4.7%, ICC = 0.96, SWC = 1.6 cm, Between; CV = 3.8%, ICC = 0.97, SWC = 1.6 cm
Heishman et al. [42]	8 female & 14 male Division 1 NCAA collegiate basketball players	CMJ (without arm swing)	Within- & between-sessions	3	CV, ICC, SWC	ToV	Within; CV = 5.4%, ICC = 0.94, SWC = 1.6 cm, Between; CV = 4.3%, ICC = 0.96, SWC = 1.6 cm
Heishman et al. [42]	8 female & 14 male Division 1 NCAA collegiate basketball players	CMJ (with arm swing)	Within- & between-sessions	3	CV, ICC, SWC	FT	Within; CV = 5.1%, ICC = 0.93, SWC = 1.8 cm, Between; CV = 3.3%, ICC = 0.97, SWC = 1.8 cm
Heishman et al. [42]	8 female & 14 male Division 1 NCAA collegiate basketball players	CMJ (with arm swing)	Within- & between-sessions	3	CV, ICC, SWC	ToV	Within; CV = 12.7%, ICC = 0.56, SWC = 2.6 cm, Between; CV = 8.6%, ICC = 0.61, SWC = 2.5 cm
Wade et al. [97]	9 females & 15 males	CMJ	Within-session	5	CV	ToV	3.8%
Wade et al. [97]	9 females & 15 males	CMJ	Within-session	5	CV	ToV+D	3.5%
Wade et al. [97]	9 females & 15 males	SJ	Within-session	5	CV	ToV	5.1%
Wade et al. [97]	9 females & 15 males	SJ	Within-session	5	CV	ToV+D	27.4%
Wade et al. [98]	11 females & 12 males	CMJ	Within-session	5	ICC	ToV+D	Maximal and submaximal jumping: 0.99
Wade et al. [98]	11 females & 12 males	CMJ	Within-session	5	ICC	ToV+D_b_	Maximal and submaximal jumping: 0.99
Pérez-Castilla et al. [80]	23 collegiate men	Loaded SJs (free weights)	Between-sessions	1	CV, ICC, SEM	FT	17 kg: CV = 8.8%, ICC = 0.76, SEM = 1.90 cm; 30 kg: CV = 8.6%, ICC = 0.82, SEM = 1.49 cm; 45 kg: CV = 7.5%, ICC = 0.89, SEM = 0.99 cm; 60 kg: CV = 15.1%, ICC = 0.78, SEM = 1.33 cm; 75 kg: CV = 19.5%, ICC = 0.74, SEM = 1.35 cm
Pérez-Castilla et al. [80]	23 collegiate men	Loaded SJs (Smith machine)	Between-sessions	1	CV, ICC, SEM	FT	17 kg: CV = 4.6%, ICC = 0.93, SEM = 0.98 cm; 30 kg: CV = 5.6%, ICC = 0.92, SEM = 0.96 cm; 45 kg: CV = 7.6%, ICC = 0.91, SEM = 0.98 cm; 60 kg: CV = 10.1%, ICC = 0.90, SEM = 0.91 cm; 75 kg: CV = 14.8%, ICC = 0.86, SEM = 0.96 cm
Pérez-Castilla et al. [80]	23 collegiate men	Loaded SJs (free weights)	Between-sessions	1	CV, ICC, SEM	ToV	17 kg: CV = 10.0%, ICC = 0.77, SEM = 2.23 cm; 30 kg: CV = 9.7%, ICC = 0.81, SEM = 1.74 cm; 45 kg: CV = 8.1%, ICC = 0.90, SEM = 1.11 cm; 60 kg: CV = 14.6%, ICC = 0.82, SEM = 1.35 cm; 75 kg: CV = 17.2%, ICC = 0.80, SEM = 1.33 cm
Pérez-Castilla et al. [80]	23 collegiate men	Loaded SJs (Smith machine)	Between-sessions	1	CV, ICC, SEM	ToV	17 kg: CV = 6.2%, ICC = 0.86, SEM = 1.41 cm; 30 kg: CV = 6.5%, ICC = 0.87, SEM = 1.27 cm; 45 kg: CV = 7.9%, ICC = 0.87, SEM = 1.28 cm; 60 kg: CV = 14.9%, ICC = 0.67, SEM = 1.82 cm; 75 kg: CV = 17.7%, ICC = 0.71, SEM = 1.89 cm
Pérez-Castilla and García-Ramos [79]	17 male physical education students	Loaded CMJs (free weights)	Between-sessions	1	CV, ICC, SEM	FT	17 kg: CV = 6.8%, ICC = 0.78, SEM = 1.71 cm; 30 kg: CV = 4.7%, ICC = 0.90, SEM = 0.97 cm; 45 kg: CV = 5.3%, ICC = 0.89, SEM = 0.85 cm; 60 kg: CV = 5.7%, ICC = 0.94, SEM = 0.62 cm; 75 kg: CV = 10.2%, ICC = 0.88, SEM = 0.88 cm
Pérez-Castilla and García-Ramos [79]	17 male physical education students	Loaded CMJs (Smith machine)	Between-sessions	1	CV, ICC, SEM	FT	17 kg: CV = 4.7%, ICC = 0.89, SEM = 1.24 cm; 30 kg: CV = 6.5%, ICC = 0.86, SEM = 1.37 cm; 45 kg: CV = 6.0%, ICC = 0.91, SEM = 0.95 cm; 60 kg: CV = 5.0%, ICC = 0.97, SEM = 0.55 cm; 75 kg: CV = 7.5%, ICC = 0.94, SEM = 0.68 cm
Pérez-Castilla and García-Ramos [79]	17 male physical education students	Loaded CMJs (free weights)	Between-sessions	1	CV, ICC, SEM	ToV	17 kg: CV = 5.8%, ICC = 0.83, SEM = 1.45 cm; 30 kg: CV = 4.9%, ICC = 0.89, SEM = 1.01 cm; 45 kg: CV = 4.3%, ICC = 0.92, SEM = 0.68 cm; 60 kg: CV = 6.7%, ICC = 0.89, SEM = 0.72 cm; 75 kg: CV = 10.4%, ICC = 0.85, SEM = 0.93 cm
Pérez-Castilla and García-Ramos [79]	17 male physical education students	Loaded CMJs (Smith machine)	Between-sessions	1	CV, ICC, SEM	ToV	17 kg: CV = 6.8%, ICC = 0.78, SEM = 1.74 cm; 30 kg: CV = 9.8%, ICC = 0.58, SEM = 2.11 cm; 45 kg: CV = 9.8%, ICC = 0.71, SEM = 1.64 cm; 60 kg: CV = 14.3%, ICC = 0.68, SEM = 1.65 cm; 75 kg: CV = 16.0%, ICC = 0.67, SEM = 1.59 cm

*CMJ* countermovement jump, *SJ* squat jump, *CV* coefficient of variation, *ICC* intra/interclass correlation coefficient, *SEM* standard error of measurement, *SWC* smallest worthwhile change, *NCAA* National Collegiate Athletic Association, $_{\textrm{b}}$ backward integration

$^{\textrm{a}}$Jumps were performed with arm swing

## Discussion

This review aims to demonstrate the effect of different equations on estimating jump height derived from CMJs, SJs, DJs, and loaded jumps using force platform data.

No consensus exists on which equation is best suited for jump height calculations using the force platform. As Table 3 shows, some studies have recommended one equation over the other, but no consistency was found. There are several equations to calculate jump height, each with advantages and disadvantages based on different assumptions and definitions, leading to confusion and inappropriate comparisons.

### Flight-Time or Take-Off Velocity?

Defining jump height as the vertical displacement of the CoM from take-off to the highest position of the CoM when in the air (from point C to point D in Fig. 1) [14, 102, 104] allows for the use of both FT and ToV for $\hbox {JH}_{\text {takeoff}}$ estimations. While ignoring pre-take-off CoM displacement (heel-rise), these equations raise questions of validity and reliability.

FT tends to overestimate $\hbox {JH}_{\text {takeoff}}$ relative to ToV by, on average, 0.6–4.1 cm across studies (Table 3). The systematic overestimation stems from the assumptions that lie in the FT equation itself. When using the time in the air to calculate $\hbox {JH}_{\text {takeoff}}$, it is assumed that the ascend time is equal to the descend time, as evident in Eq. (11) [102]. This assumption rarely holds true as participants are likely to bend their legs slightly while in the air, which extends the descend and artificially increases flight time and, thus, $\hbox {JH}_{\text {takeoff}}$ (when calculated from Eq. (14)) [1, 4, 18, 50, 69, 86, 97–99, 102–104].

The joint bending phenomenon has been proposed to be an unconscious preparation for the large landing forces, with some clear individual differences [4, 49, 98, 103]. Instructions to maintain leg extension upon landing can mitigate this effect [5, 79, 80], but on the downside, instructions on how to land may compromise the push-off and result in submaximal jump heights [4, 103]. Therefore, some investigators/coaches may be unwilling to instruct their participants or athletes to land in a specific manner, when they are interested in maximum jump height. Indeed, only one of the studies in Table 3, using FT for unloaded jumps, reported giving their participants specific landing instructions [103].

We are not aware of any studies that have investigated whether jump height (performance) per se affects the landing technique, but Haug et al. [38] observed a clear difference in the height of the CoM at take-off in nonathletes, short track speed skaters, and weightlifters. The weightlifters that jumped the highest had about 3 cm less elevation of the CoM at take-off compared with the nonathletes in the SJ. This may be due to a more premature take-off [11] and less lower-body joint extension in the weightlifters who reached more than 20% higher peak velocity of the CoM during the push-off. With a similar landing (slightly bent legs), the FT might give more or less valid results for the two groups.

#### Reliability Considerations

The choice between FT and ToV for estimating $\hbox {JH}_{\text {takeoff}}$ is a matter not only of agreement between equations but also of their suitability for comparing performance within and across individuals, both within and between sessions.

From Table 4, it seems that the reliability measures are comparable between ToV and FT or, in fact, better for FT (ToV shows 0.3–9.3% larger coefficient of variation (CV)—i.e., worse reliability—compared with FT for the majority of the results). The better reliability of $\hbox {JH}_{\text {takeoff}}$ when using FT compared with ToV is in contrast to other findings [102]. However, better reliability for FT could be attributed to the fact that FT only relies on the time at take-off and landing to estimate $\hbox {JH}_{\text {takeoff}}$. ToV, on the other hand, requires accurate body weight measures, detecting the correct start of integration and take-off, where the integration procedure could be affected by drift in the GRF–time signals. It is crucial to understand that such discrepancies are not inherent to the jump height equations themselves but rather stem from suboptimal execution by the user and the properties of the equipment. Thus, when interpreting the reliability results discussed above, one must consider the proficiency with which each equation is applied. As the accuracy of the GRF–time data processing steps must be considered, it is important to note that ToV requires more data processing, which could contribute to decreased reliability compared with FT. To exemplify, Heishman et al. [42] reported worse reliability for $\hbox {JH}_{\text {takeoff}}$ calculated by ToV for jumps performed with arm swings than jumps performed without arm swings (akimbo), which was most likely owing to more noisy GRF–time data in the jumps with arm swings than akimbo.

The CV (a normal measure of reliability) is, however, not only determined by error in the calculation procedures. The CV will also be affected by biological variation and error attributed to poor test execution (e.g., test instructions). The biological variation should be the same for FT and ToV but can be larger for FT if the athletes are not used to jumping. FT also relies on using the most exact test instructions, since FT is affected by the landing technique. Nonetheless, similar take-off and landing strategies cannot be guaranteed even for the same individual, especially if jumps with different external loads are used. Fatigue and recovery status may also affect both jumping and landing strategies [31, 43], and this should be considered when testing the same individual at multiple time points, e.g., during an athlete’s season.

Overall, ToV is used more than FT when calculating jump height using the force platform, especially in research settings [9, 22, 34, 35, 39–41, 47, 52, 55, 57, 64, 66, 85, 88]. It is not clear whether this is due to tradition and the belief that the ToV is superior to FT, or if it reflects that ToV is truly better than FT. To ensure both validity and reliability, reporting both ToV and FT could be good practice if a force plate is used.

#### What about Loaded Jumps?

Consistent with unloaded jumps, investigations of loaded CMJs and SJs indicate that FT might offer more reliable measurements than ToV (Table 4). Following these lines, Pérez-Castilla et al. [79, 80] concluded that FT provided more reliable measures of Smith-machine-loaded CMJs and SJs than ToV (Tables 3 and 4).

The constrained movement path of the Smith machine likely contributes to the increased reliability by standardizing the jump technique, thus reducing the intrinsic errors associated with FT discussed above. However, Pérez-Castilla et al. [79, 80] discussed how frictional forces from the linear bearings of the Smith machine increase the propulsion time and thereby artificially increase the net vertical impulse of SJs. This results in overestimated $\hbox {JH}_{\text {takeoff}}$ when estimated by ToV compared with FT, an effect that may become more pronounced with heavier loads (Table 3). Interestingly, the proposed friction effect was not observed for Smith-machine-loaded CMJs, where the static friction probably is overcome during the countermovement phase. We would, however, suggest that the friction effect may vary between equipment, and care should be taken when comparing jump heights between Smith machines.

For free-weight barbell jumps, FT and ToV demonstrated similar $\hbox {JH}_{\text {takeoff}}$ and reliability for both SJs and CMJs (Tables 3 and 4; [79, 80]). Landing technique instructions could have influenced these outcomes [80]. However, Lindberg et al. [54] demonstrated FT to overestimate loaded $\hbox {JH}_{\text {takeoff}}$, compared with ToV, even when clear landing instructions were given. The discrepancies in these findings suggest that calculation protocols and athlete performance levels, which differed in these studies, may also affect jump height estimations.

In conjunction with Pérez-Castilla et al. [79, 80], Lindberg et al. [54] reported that FT demonstrated better between-session reliability compared with ToV, during loaded jumps (additional data were provided by the authors). Lindberg et al. [54] highlighted potential difficulties in detecting jump phases that are required when using ToV owing to noisy GRF–time data, especially for loaded SJs. Therefore, both Pérez-Castilla et al. [79, 80] and Lindberg et al. [54] recommended choosing FT over ToV when assessing $\hbox {JH}_{\text {takeoff}}$ from loaded CMJs and SJs, both in a Smith machine and when using free weights.

It should be recognized that adding external loads to a jumper results in a system (body plus additional load) with a higher CoM position than an unloaded body. Equivalent joint positions and movements will thus result in greater sagittal plane excursion of the CoM with heavier loads. This may alter the neuromuscular demands of jumping and require different jump strategies at light and heavy loads. Most prominently, jumping with external loads increases the relative hip joint contribution and reduces the knee joint contribution [70]. External loads will also slow the push-off movement and allow for more complete extension of the ankle, knee, and hip joints at take-off [11]. The more extended position in the take-off might facilitate a more extended position in the landing, especially considering the short flight time, which may explain why the FT method is more appropriate for loaded jumps. The kinematics of landing from maximal CMJs and SJs warrants investigation to support this point.

### Including the Heel-Rise

While practitioners and researchers might consider FT or ToV to be satisfactory for jump height calculations, others have stated that jump height is affected by both the velocity and the position of the CoM at take-off, as simply estimating jump height from toe-off neglects the change in CoM position between standing and take-off (i.e., heel-rise; point A to C in Fig. 1) [18, 69, 99, 102, 104].

Both FT and ToV give lower jump heights compared with ToV+D and DIS by up to $\sim$ 15 cm (Table 3), because they neglect the displacement of the CoM prior to take-off. In the studies where ToV+D or DIS were recommended over ToV or FT (Table 3; [18, 86, 99]), the conclusions were based on the fact that ToV+D or DIS provided $\hbox {JH}_{\text {standing}}$ values closer in magnitude to a 3D motion capture criterion, i.e., the difference in CoM position taken from standing until the apex of the jump (from point A to point D in Fig. 1).

With regard to the FT approach, Wade et al. [97] proposed a method to improve the FT equation for calculating $\hbox {JH}_{\text {standing}}$, where an anthropometrically scaled constant accounting for the heel-rise was used. However, even when accounting for the heel-rise, there may be inconsistent landing techniques, which would influence the FT values [98].

The fact that ToV+D and DIS provide higher jump heights than ToV or FT does not indicate that these equations are better for jump height estimations. ToV+D and DIS are simply defining jump height differently; i.e., ToV+D and DIS account for the heel-rise prior to take-off, while ToV and FT do not [102, 104]. Which one of these jump height definitions is the “correct” one remains debatable. Perhaps including heel-rise could better represent the specific biomechanics of the jump and might be useful when there is an interest in jumping mechanisms [10, 11]. Notably, the work done on the CoM, from standing to the apex of the jump (between points A and D in Fig. 1), is only captured by ToV+D and DIS. Not including heel-rise will fail to account for the work required to raise the CoM higher for an athlete with larger feet than someone with smaller feet. Perhaps most importantly, ToV+D and DIS represent the absolute displacement of the CoM over the ground and are, thus, more representative of how high an athlete can jump; e.g., imagine two basketball players dueling to reach the ball in the air. In contrast, the ToV or FT represents a displacement of the CoM from an unknown take-off point, which can vary between individuals [38, 103].

Nevertheless, not including heel-rise for jump height calculations is a more straightforward approach that provides a simpler estimation of jump height and should be sufficient in a sporting context [102], if comparing jump height results within an athlete over time. Moreover, from a technical perspective, the fact that ToV+D and DIS require double integration of the GRF–time data renders them more prone to integration drifts, and thus, they might not be suitable in all instances.

#### Integration Drifts

For $\hbox {JH}_{\text {standing}}$, DIS has only been recommended when not compared with ToV+D (Table 3). In the one study comparing ToV+D and DIS, even though both equations provided $\hbox {JH}_{\text {standing}}$ estimates closer to a 3D motion criterion (compared with $\hbox {JH}_{\text {takeoff}}$), Chiu and Dæhlin [18] recommended ToV+D over DIS owing to larger random errors observed for the latter. The larger random error observed for DIS compared with ToV+D was suggested to mirror the integration of the force–time signal in the flight phase of the jump. Ideally, the force platform would register zero force during the flight phase, but drift in raw force signals can introduce nonzero readings, impacting the double integration necessary when using DIS for $\hbox {JH}_{\text {standing}}$ estimations [18]. One suggestion is to subtract the force data extracted during the flight phase from the total GRF–time data to achieve a near-zero GRF–time signal for the flight phase. However, even with a zero force trace during the flight phase, the time required for integration in ToV+D and DIS will result in integration drifts. While it is possible to control for drift, this is not practical for in-field applications. Moreover, drift is mainly a concern if users intend to process the GRF–time data themselves, using tools such as Excel or coding languages. For some proprietary software, users cannot account for drift. Nevertheless, both practitioners and researchers should consider this point when interpreting jump height results obtained from ToV+D and DIS.

Chiu and Dæhlin [18] analyzed CMJs and suggested that ToV+D and DIS were not suited for estimating SJ or DJ heights. Wade et al. [97] observed how ToV+D provided accurate $\hbox {JH}_{\text {standing}}$ estimates for the CMJ, although they advised against using this equation for SJ height calculations, owing to larger integration drift risks (integrating the GRFs from the upraised standing position, through the squat position (held for 1–2 s)—increasing integration time). Indeed, Wade et al. [97] reported how double integration of the force data resulted in clear errors in jump height and negative values for heel-lift, which has not been reported in other studies or by methods not including the force data. Conversely, Wank and Coenning [99] recommended DIS over ToV and FT for SJ height estimates (Table 3), which was based on the fact that DIS provided SJ heights closer in magnitude to a 3D motion criterion. A clear difference between the studies by Wade et al. [97] and Wank and Coenning [99] is how the latter started integration from the end of the jump (performing a backward integration procedure), while Wade et al. [97] started integration from the beginning of the jump. Indeed, backward integration could offset the limitations mentioned by Wade et al. [97], as it would require a shorter integration time for SJ height calculations since the squat position is not held after landing. However, a major limitation of using backward integration procedures is the time required to achieve a standing still position, especially for inexperienced participants. Moreover, slight differences in the body weight measurements from before to after the jump could affect jump height when performed via backward integration procedures.

#### Criterion Dependence

All studies that have recommended ToV+D or DIS over FT or ToV have used 3D motion capture as a criterion, where jump height has been defined from point A to point D in Fig. 1 ($\hbox {JH}_{\text {standing}}$). Moir [69] did not include 3D motion capture, where ToV+D was the criterion, resulting in the recommendation of ToV over both ToV+D and FT (Table 3). Moir [69] observed that ToV+D provided little additional information compared with ToV as the CoM displacement at take-off was not significantly different between a group of men and women, while jump height was significantly higher in the group of men, indicating that the latter was due to differences in take-off velocity. These results agreed with previous findings [2] but have not been supported by newer findings [18]. The question is whether we should be concerned with the equation best suited for comparing jump heights both within and between participants or which equation gives values closer to an assumed true jump height.

### Drop Jump Analyses

As described in Sect. 1.2 and illustrated in Fig. 3, four different methods can be used to calculate DJ height using force platforms. One of these methods requires two force platforms, while the remaining three methods require a single force platform.

On the basis of the studies presented in Table 3, the double force platform method is generally recommended over any of the single force platform methods for calculating DJ heights. This is simply because the double force platform method provides the most direct estimate of the initial velocity condition (Eq. 4) required if using ToV for $\hbox {JH}_{\text {takeoff}}$ estimates.

However, it is crucial to consider the exponential increase in the use of portable force platforms for in-field settings [65], and that many practitioners are unlikely to possess two force platforms [50, 65]. Furthermore, most practitioners rely on commercial software that does not calculate DJ height using the double force platform method. Consequently, in practical applications, any of the single force platform methods will be employed.

When using a single force platform for DJ analysis, three different methods can be employed: ToV estimating touchdown velocity from box height, ToV using backward integration, or FT (Fig. 3).

#### Box Height Method

As seen in Table 3, the box height method has been advised against for DJ analysis. The reason is that the box height does not represent the true drop height of the participant. Indeed, research highlights how participants tend to elevate their CoM when dropping from low box heights (20 cm) while lowering their CoM when dropping from higher box heights (40–60 cm), which in turn would lead to the box height under- (at low box heights) or overestimating (at higher box heights) actual drop height [12, 50]. However, it has also been reported that drop height is overestimated at low box heights (20 cm) by up to $\sim$ 5 cm [23]. Nonetheless, to the best of our knowledge, all studies that have investigated this phenomenon have reported how actual drop height differs from box height by, on average, 0.7–12.5 cm, where the absolute difference increases with higher box heights [12, 20, 23, 50, 65]. It is noteworthy that some commercial software, which most practitioners rely on, calculates DJ heights by using box height as an input for estimating touchdown velocity [9, 57]. Practitioners should be careful when interpreting DJ results from proprietary software.

#### Backward Integration Method

The backward integration method has been reported by Baca [4] to result in better accuracy for $\hbox {JH}_{\text {takeoff}}$ compared with using the box height or FT methods [4], while Jørgensen et al. [46] observed how the backward integration approach, using a single force platform, resulted in greater intraindividual variability in $\hbox {JH}_{\text {takeoff}}$ compared with the reference double force platform method.

Neither Jørgensen et al. [46] nor Baca [4] recommended the backward integration method for DJ analysis (for estimating $\hbox {JH}_{\text {takeoff}}$).

Baca [4] argued against using ToV with backward integration owing to the difficulty of having participants land on the force platform and remaining still, leading to many repeated trials. Baca [4] further discussed how the requirement to stand still after the jump could influence the jump technique and, thus, performance.

McMahon et al. [65] suggested a proposed method where any discrepancies between the velocity obtained from the average final second of data recording and the estimated touchdown velocity derived from the box height were used as a correction factor for the latter. McMahon et al. [65] observed that using the proposed method with a single force platform provided equally accurate drop height and touchdown velocity estimates, compared with the double force platform method. Consequently, the proposed method resulted in equally accurate $\hbox {JH}_{\text {takeoff}}$ measures when using the corrected touchdown velocity as the initial velocity (Table 3).

In contrast to the report by Baca [4], McMahon et al. [65] did not encounter challenges with their participants landing on the force platform and remaining still for the final second of data recording. The discrepancies in these observations can be attributed to the fact that McMahon et al. [65] instructed their participants to perform a controlled landing and remain still until the end of data recording, while Baca [4] simply instructed participants to “jump as high as possible” (though excluding jumps where the participants failed to land on the force platform). Jørgensen et al. [46] also instructed participants to reach a still position for 2 s as fast as possible after landing. Wank and Coenning [99] also recommended estimating DJ heights ($\hbox {JH}_{\text {standing}}$) via backward integration procedures, although they did not mention the instructions given or directly compare this approach with other alternatives. On the basis of the experimental findings, force data from the end of the jump can only be used if participants have been instructed and are able to land on the force platform and remain still to ensure accurate body weight and integration procedures [59]. The achievability of these requirements likely depends on factors such as the size of the force platform, the height of the box from which the person drops, the height of the jump, the experience of the jumper, the willingness to give landing instructions when testing maximal jump heights, etc.

#### Flight-Time Method

If the landing criterion is not met, it is interesting to note that, in Baca [4], FT showed better agreement with the double force platform method for DJ height ($\hbox {JH}_{\text {takeoff}}$) estimates compared with using the estimated touchdown velocity from box height (Table 3). Although not recommended, FT was considered useful when comparing trials within the same participant or athlete [4]. Thus, if data from the end of the jump cannot be used, FT (with its limitations highlighted in Sect. 4.1) might be the best secondary option for DJ height ($\hbox {JH}_{\text {takeoff}}$) calculations using a single force platform.

## Practical Applications

The purpose of this review is to inform researchers and practitioners about the expected differences in jump height and the reasons for these, considering the different equations that can be chosen for jump height estimations using force platforms.

It is strongly advised that anyone using force platforms to estimate jump height considers which equation should be employed in advance of their testing based on:

(i) The jump type tested
(ii) The reason for testing
(iii) How one defines jump height

**Fig. 5:**
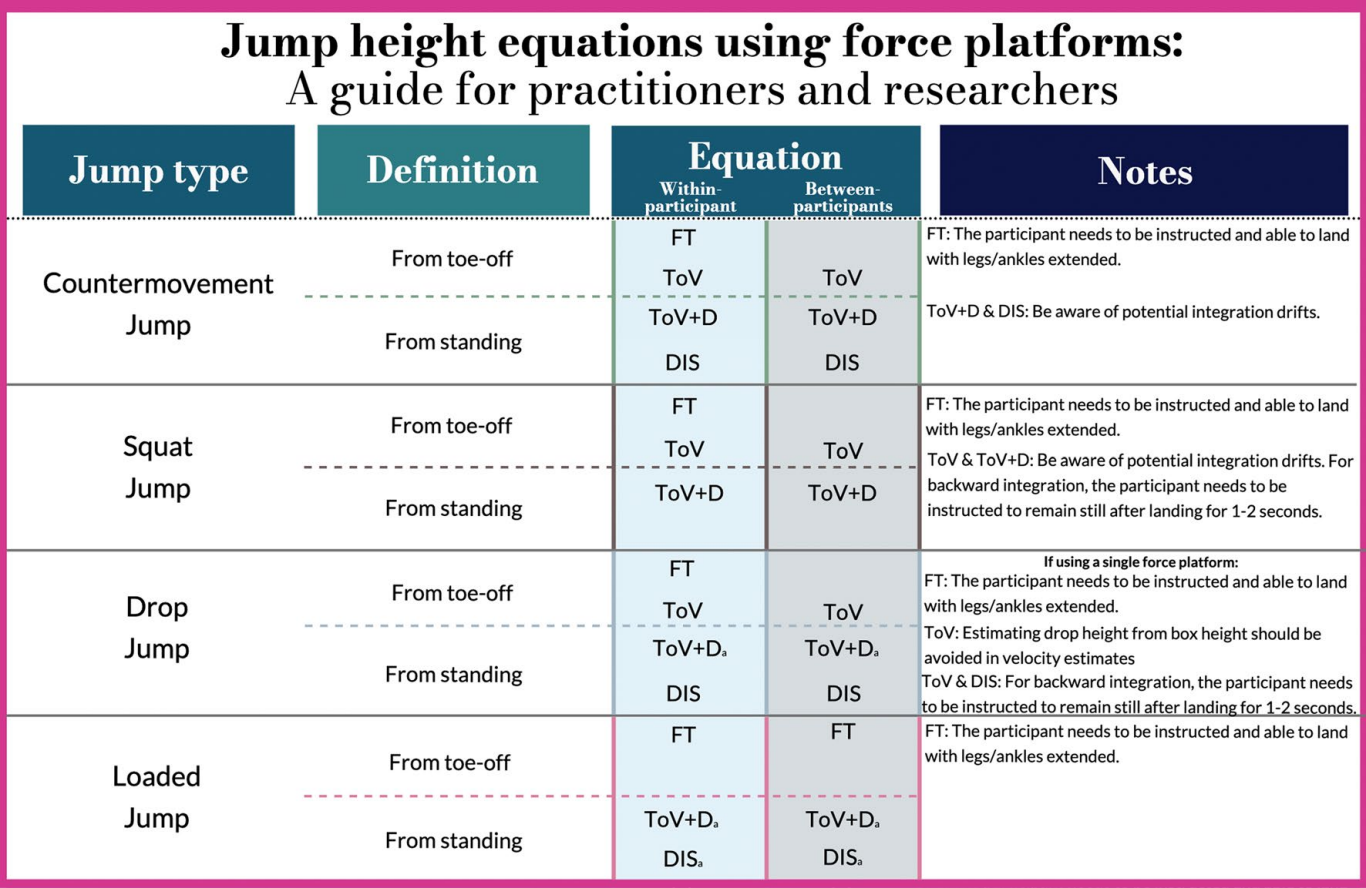
A map of considerations to be taken into account when choosing a jump height equation using force platforms. First, one should consider the jump modality tested. Second, one should choose the definition of jump height that is suited to the context. Third, the jump height equations are chosen, but the decision must account for the reason for testing, i.e., whether the results are used for within- or between-participants comparisons. If the equation is not presented, it has not been recommended for the intended use. Lastly, regardless of the equation chosen, there are considerations that should be accounted for, which are indicated in the Notes section. ^a^No research has examined the suitability of these equations for the respective jump modality

These points are summarized in Fig. 5, which outlines a map of what one should consider with regard to equation choices when estimating jump height from force platforms.

First and foremost, it is important to establish a clear definition of jump height. While a common and simple approach is to define jump height without accounting for heel-rise, either by ToV or FT, incorporating the heel-rise (via ToV+D or DIS) may be important when testing for performance implications. Nevertheless, regardless of the definition employed, certain technical aspects that depend on the type of jump being tested and the purpose of the test must be considered.

FT can be used to compare $\hbox {JH}_{\text {takeoff}}$ within participants for CMJs, SJs, and DJs, but it is imperative to ensure fully extended lower limbs in the landing. This requires specific landing instructions and experienced athletes. Owing to individual differences in landing technique, the FT equation is generally not recommended for between-participant comparisons. However, for loaded CMJs and SJs, FT may be preferred over any of the impulse–momentum equations.

Access to equipment is a major factor contributing to the equation used to assess jump height, as addressed by Xu et al. [102]. In many cases, a force platform is not accessible, and FT is the only option to calculate jump height via contact mats or smartphones [102]. Thus, when estimating $\hbox {JH}_{\text {takeoff}}$ from FT, the results could be more easily compared with others [1]. The advantage of using a force plate is then that other variables, such as maximal force, rate of force development, and peak power, can be used to evaluate the neuromuscular condition of an athlete [31, 43].

ToV+D has often been recommended in the literature over ToV or FT for CMJ height estimates, because ToV+D includes the heel-rise, while ToV and FT do not. It is worth noting that these recommendations stem from studies using 3D motion capture as the criterion, where jump height has been defined as $\hbox {JH}_{\text {standing}}$ (from point A to point D in Fig. 1). DIS defines jump height closest to a jump height definition of 3D motion capture. From an applied sports context, it may also seem obvious to estimate jump height as the difference of the position of the CoM from standing until the highest point in the air (consider two basketball players dueling to reach the ball in the air). However, ToV+D and DIS require more data processing than ToV or FT, where DIS requires double integration of the GRF–time data over the longest time period compared with the other equations. Double integration of the GRF–time data can lead to drift, which increases with time and may cause errors in $\hbox {JH}_{\text {standing}}$ estimates. In fact, this may make DIS unsuitable in some cases, especially for SJs if applying a forward integration procedure. Therefore, if disregarding the jump height definition, it is not necessarily clear that ToV+D and DIS are better options for jump height estimates compared with, e.g., ToV, which is robust for all jumping modalities.

Given that differences in jump height of up to $\sim$ 15 cm have been reported when accounting for the heel-rise versus not, one cannot compare jump heights estimated by ToV or FT with those estimated by ToV+D or DIS.

When testing DJs with a single force platform, instructions on how to land are necessary as estimating drop height from the box height should be avoided. If planning to apply a backward integration procedure, the participants must be instructed to land on the force platform and come to a still position as quickly as possible. Using the FT equation might be the second-best option if working with a single force platform, where the participants must then be instructed to land with the legs extended. These points are important for anyone analyzing DJs, as it is necessary to plan what instructions should be given (on the basis of the equation used) before testing. If two force platforms are available, the double force platform method is recommended rather than using any of the single force platform methods.

## Conclusions

Four equations have been commonly described in the literature for jump height estimates using the force platform: FT, ToV, ToV+D, and DIS—where the two latter methods include the heel-rise, while the two former methods do not. ToV has been the most recommended equation and has typically been used as a reference equation in methodological studies for jump height calculations. However, most of the current recommendations on which of these jump height equations to apply do not account for factors such as different jump height definitions, landing instructions, ease of use in practical settings, and/or data processing. We claim, however, that these are important considerations. Which of the four equations is best suited for jump height estimates depends on (i) the jump modality tested, (ii) the reason for testing, and (iii) how one defines jump height. None of the equations should be used interchangeably, but they may be presented side by side or as supplementary data to be used and compared with other data. The findings from this review highlight the advantages and disadvantages of the different jump height equations and provide recommendations for selecting the most appropriate equation on the basis of the specific setting.

Without the context of the method applied, jump heights cannot be compared with those obtained by others.

## References

[1] Aragón LF. Evaluation of four vertical jump tests: methodology, reliability, validity, and accuracy. Meas Phys Educ Exerc Sci. 2000;4(4):215–28.

[2] Aragón-Vargas LF, Gross MM. Kinesiological factors in vertical jump performance: differences within individuals. Occup Health Ind Med. 1997;13(1):45–65.

[3] Asmussen E, Bonde-petersen F. Storage of elastic energy in skeletal muscles in man. Acta Physiol Scand. 1974;91(3):385–92. 4846332 10.1111/j.1748-1716.1974.tb05693.x

[4] Baca A. A comparison of methods for analyzing drop jump performance. Med Sci Sports Exerc. 1999;31(3):437–42. 10188749 10.1097/00005768-199903000-00013

[5] Balsalobre-Fernández C, Glaister M, Lockey RA. The validity and reliability of an iphone app for measuring vertical jump performance. J Sports Sci. 2015;33(15):1574–9. 25555023 10.1080/02640414.2014.996184

[6] Barker L, Harry JR, Dufek JS, Mercer J. Aerial rotation effects on vertical jump performance among highly skilled collegiate soccer players. J Strength Cond Res. 2017;31:932–8. 27398922 10.1519/JSC.0000000000001557

[7] Barr M, Nolte VW. Which measure of drop jump performance best predicts sprinting speed? J Strength Cond Res. 2011;25(7):1976–82. 21701285 10.1519/JSC.0b013e3181e4f7ba

[8] Beckham TS, Mizuguchi GS. Force plate use in performance monitoring and sport science testing. New Stud Athlet. 2014;29(3):25–37.

[9] Bishop C, Pereira LA, Reis VP, Read P, Turner AN, Loturco I. Comparing the magnitude and direction of asymmetry during the squat, countermovement and drop jump tests in elite youth female soccer players. J Sports Sci. 2020;38(11–12):1296–303. 31354103 10.1080/02640414.2019.1649525

[10] Bobbert MF, van Ingen Schenau GJ. Coordination in vertical jumping. J Biomech. 1988;21(3):249–62. 3379084 10.1016/0021-9290(88)90175-3

[11] Bobbert MF, van Soest AJ. Why do people jump the way they do? Exerc Sport Sci Rev. 2001;29(3):95–102. 11474963 10.1097/00003677-200107000-00002

[12] Bobbert MF, Mackay M, Schinkelshoek D, Huijing P, van Ingen Schenau G. Biomechanical analysis of drop and countermovement jumps. Eur J Appl Physiol Occup Physiol. 1986;54:566–73. 3948851 10.1007/BF00943342

[13] Bosco C, Komi PV. Potentiation of the mechanical behavior of the human skeletal muscle through prestretching. Acta Physiol Scand. 1979;106(4):467–72. 495154 10.1111/j.1748-1716.1979.tb06427.x

[14] Bosco C, Luhtanen P, Komi PV. A simple method for measurement of mechanical power in jumping. Eur J Appl Physiol. 1983;50:273–82. 10.1007/BF00422166 6681758

[15] Buckthorpe M, Morris J, Folland JP. Validity of vertical jump measurement devices. J Sports Sci. 2012;30(1):63–9. 22111944 10.1080/02640414.2011.624539

[16] Castagna C, Ganzetti M, Ditroilo M, Giovannelli M, Rocchetti A, Manzi V. Concurrent validity of vertical jump performance assessment systems. J Strength Cond Res. 2013;27(3):761–8. 22648140 10.1519/JSC.0b013e31825dbcc5

[17] Chavda S, Bromley T, Jarvis P, Williams S, Bishop C, Turner AN, Lake JP, Mundy PD. Force-time characteristics of the countermovement jump: analyzing the curve in excel. Strength Cond J. 2017;40(2):67–77.

[18] Chiu LZF, Dæhlin TE. Comparing numerical methods to estimate vertical jump height using a force platform. Meas Phys Educ Exerc Sci. 2020;24(1):25–32.

[19] Cormack SJ, Newton RU, McGuigan MR, Doyle TL. Reliability of measures obtained during single and repeated countermovement jumps. Int J Sports Physiol Perform. 2008;3(2):131–44. 19208922 10.1123/ijspp.3.2.131

[20] Costley L, Wallace ES, Johnston MJ, Kennedy RA. Reliability of bounce drop jump parameters within elite male rugby players. J Sports Med Phys Fitness. 2018;58(10):1390–7. 28745469 10.23736/S0022-4707.17.07400-X

[21] Cronin JB, Hing RD, McNair PJ. Reliability and validity of a linear position transducer for measuring jump performance. J Strength Cond Res. 2004;18(3):590–3. 15320688 10.1519/1533-4287(2004)18<590:RAVOAL>2.0.CO;2

[22] Cross MR, Rivière JR, Van Hooren B, Coulmy N, Jiménez-Reyes P, Morin J-B, Samozino P. The effect of countermovement on force production capacity depends on extension velocity: a study of alpine skiers and sprinters. J Sports Sci. 2021;39(16):1882–92. 33792497 10.1080/02640414.2021.1906523

[23] de Fátima Geraldo G, da Glória TelesBredt S, Menzel HJ, da Cunha Peixoto Cançado GH, de Carvalho LC, Lima FV, Soares JAS, de Andrade AGP. Drop height is influenced by box height but not by individual stature during drop jumps a altura da queda e influeniada pela altura da caixa, mas nao pela estatura individiaul durante os saltos em profundidade. J Phys Educ. 2019;30:3078.

[24] Dias JA, Dal Pupo J, Reis DC, Borges L, Santos SG, Moro AR, Borges NG Jr. Validity of two methods for estimation of vertical jump height. J Strength Cond Res. 2011;25(7):2034–9. 21701288 10.1519/JSC.0b013e3181e73f6e

[25] Donahue PT, Hill CM, Wilson SJ, Williams CC, Garner JC. Squat jump movement onset thresholds influence on kinetics and kinematics. Int J Kinesiol Sports Sci. 2021;9(3):1–7.

[26] Dowling JJ, Vamos L. Identification of kinetic and temporal factors related to vertical jump performance. J Appl Biomech. 1993;9(2):95–110.

[27] Farris DJ, Lichtwark GA, Brown NAT, Cresswell AG. The role of human ankle plantar flexor muscle-tendon interaction and architecture in maximal vertical jumping examined in vivo. J Exp Biol. 2016;219(4):528–34. 26685172 10.1242/jeb.126854

[28] Gallardo-Fuentes F, Gallardo-Fuentes J, Ramirez-Campillo R, Balsalobre-Fernández C, Martínez C, Caniuqueo A, Cañas R, Banzer W, Loturco I, Nakamura FY, Izquierdo M. Intersession and intrasession reliability and validity of the my jump app for measuring different jump actions in trained male and female athletes. J Strength Cond Res. 2016;30(7):2049–56. 27328276 10.1519/JSC.0000000000001304

[29] García-Ramos A, Feriche B, Pérez-Castilla A, Padial P, Jaric S. Assessment of leg muscles mechanical capacities: Which jump, loading, and variable type provide the most reliable outcomes? Eur J Sport Sci. 2017;17(6):690–8. 28338423 10.1080/17461391.2017.1304999

[30] García-Ramos A, Janicijevic D, Cobo-Font J, Marcos-Frutos D, Fernandes JFT, Taube W, Pérez-Castilla A. Knowledge of results during vertical jump testing: an effective method to increase the performance but not the consistency of vertical jumps. Sports Biomech. 2020;22(7):798–810. 32564674 10.1080/14763141.2020.1764090

[31] Gathercole RJ, Sporer BC, Stellingwerff T, Sleivert GG. Comparison of the capacity of different jump and sprint field tests to detect neuromuscular fatigue. J Strength Cond Res. 2015;29(9):2522–31. 26308829 10.1519/JSC.0000000000000912

[32] Glatthorn JF, Gouge S, Nussbaumer S, Stauffacher S, Impellizzeri FM, Maffiuletti NA. Validity and reliability of optojump photoelectric cells for estimating vertical jump height. J Strength Cond Res. 2011;25(2):556–60. 20647944 10.1519/JSC.0b013e3181ccb18d

[33] Harman EA, Rosenstein MT, Frykman P, Rosenstein RM. The effects of arms and countermovement on vertical jumping. Med Sci Sports Exerc. 1990;22(6):825–33. 2287261 10.1249/00005768-199012000-00015

[34] Harry JR. Matlab guide for analyzing countermovement jump strategies and performance over time. Strength Cond J. 2021;43(5):44–53.

[35] Harry JR, Barker LA, James R, Dufek JS. Performance differences among skilled soccer players of different playing positions during vertical jumping and landing. J Strength Cond Res. 2018;32(2):304–12. 29369951 10.1519/JSC.0000000000002343

[36] Harry JR, Barker L, Paquette MR. A joint power approach to define countermovement jump phases using force platforms. Med Sci Sports Exerc. 2020;52(4):993–1000. 31688643 10.1249/MSS.0000000000002197

[37] Harry JR, Blinch J, Barker L, Krzyszkowski J, Chowning LD. Low-pass filter effects on metrics of countermovement vertical jump performance. J Strength Cond Res. 2020;36(5):1459–67. 32287092 10.1519/JSC.0000000000003611

[38] Haug WB, Spratford W, Williams KJ, Chapman DW, Drinkwater EJ. Differences in end range of motion vertical jump kinetic and kinematic strategies between trained weightlifters and elite short track speed skaters. J Strength Cond Res. 2015;29(9):2488–96. 25774628 10.1519/JSC.0000000000000889

[39] Haugen T, Paulsen G, Seiler S, Sandbakk O. New records in human power. Int J Sports Physiol Perform. 2018;13(6):678–86. 28872385 10.1123/ijspp.2017-0441

[40] Haugen TA, Breitschädel F, Wiig H, Seiler S. Countermovement jump height in national-team athletes of various sports: a framework for practitioners and scientists. Int J Sports Physiol Perform. 2020;16(2):184–9. 33217727 10.1123/ijspp.2019-0964

[41] Haugen TA, Hopkins W, Breitschädel F, Paulsen G, Solberg PA. Fitness tests and match performance in a male ice hockey national league. Int J Sports Physiol Perform. 2021;16(9):1303–10. 33662926 10.1123/ijspp.2020-0644

[42] Heishman AD, Daub BD, Miller RM, Freitas EDS, Frantz BA, Bemben MG. Countermovement jump reliability performed with and without an arm swing in ncaa division 1 intercollegiate basketball players. J Strength Cond Res. 2020;34(2):546–58. 30138237 10.1519/JSC.0000000000002812

[43] Helland C, Midttun M, Saeland F, Haugvad L, Olstad DS, Solberg PA, Paulsen G. A strength-oriented exercise session required more recovery time than a power-oriented exercise session with equal work. PeerJ. 2020;8: e10044. 33062443 10.7717/peerj.10044 PMC7532781

[44] Jiménez-Reyes P, Samozino P, Cuadrado-Peñafiel V, Conceição F, González-Badillo JJ, Morin J-B. Effect of countermovement on power-force-velocity profile. Eur J Appl Physiol. 2014;114:2281–8. 25048073 10.1007/s00421-014-2947-1

[45] Jiménez-Reyes P, Samozino P, Pareja-Blanco F, Conceição F, Cuadrado-Peñafiel V, González-Badillo JJ, Morin J-B. Validity of a simple method for measuring force-velocity-power profile in countermovement jump. Int J Sports Physiol Perform. 2017;12(1):36–43. 27002490 10.1123/ijspp.2015-0484

[46] Jørgensen SL, Bojsen-Møller J, Skalgard T, Olsen HB, Aagaard P. Dual vs single force plate analysis of human drop jumping. Transl Sports Med. 2021;4(5):637–45.

[47] Katsikari K, Bassa E, Skoufas D, Lazaridis S, Kotzamanidis C, Patikas DA. Kinetic and kinematic changes in vertical jump in prepubescent girls after 10 weeks of plyometric training. Pediatr Exerc Sci. 2020;32(2):81–8. 31958772 10.1123/pes.2019-0188

[48] Kennedy RA, Drake D. Improving the signal-to-noise ratio when monitoring countermovement jump performance. J Strength Cond Res. 2018;35(1):85–90. 10.1519/JSC.0000000000002615 29742747

[49] Kibele A. Possibilities and limitations in the biomechanical analysis of countermovement jumps: a methodological study. J Appl Biomech. 1998;14(1):105–17.

[50] Kibele A. Technical note. Possible errors in the comparative evaluation of drop jumps from different heights. Ergonomics. 1999;42(7):1011–4. 10424188 10.1080/001401399185270

[51] Kons RL, Ache-Dias J, Detanico D, Barth J, Pupo JD. Is vertical jump height an indicator of athletes’ power output in different sport modalities? J Strength Cond Res. 2018;32(3):708–15. 29466272 10.1519/JSC.0000000000001817

[52] Kozinc Žitnik J, Smajla D, Šarabon N. The difference between squat jump and countermovement jump in 770 male and female participants from different sports. Eur J Sport Sci. 2022;22(7):985–93. 34075858 10.1080/17461391.2021.1936654

[53] Lake JP, McMahon JJ. Within-subject consistency of unimodal and bimodal force application during the countermovement jump. Sports. 2018;6(4):143. 30413012 10.3390/sports6040143 PMC6316337

[54] Lindberg K, Solberg PA, Bjørnsen T, Helland C, Rønnestad BR, Frank MT, Haugen TA, Østerås S, Kristoffersen MB, Midttun M, Sæland FO, Paulsen G. Force-velocity profiling in athletes: reliability and agreement across methods. PLoS ONE. 2021;16(2): e0245791. 33524058 10.1371/journal.pone.0245791 PMC7850492

[55] Lindberg K, Solberg P, Bjørnsen T, Helland C, Rønnestad B, Frank MT, Haugen T, Østerås S, Kristoffersen M, Midttun M, Sæland F, Eythorsdottir I, Paulsen G. Strength and power testing of athletes: a multicenter study of test-retest reliability. Int J Sports Physiol Perform. 2022;17(7):1103–10. 35477896 10.1123/ijspp.2021-0558

[56] Linthorne NP. Analysis of standing vertical jumps using a force platform. Am J Phys. 2001;69(11):1198–204.

[57] Makaracı Y, Özer Ö, Soslu R, Uysal A. Bilateral counter movement jump, squat, and drop jump performances in deaf and normal-hearing volleyball players: a comparative study. J Exerc Rehab. 2021;17(5):339. 10.12965/jer.2142522.261 PMC8566106 34805023

[58] Malisoux L, Gette P, Urhausen A, Bomfim J, Theisen D. Influence of sports flooring and shoes on impact forces and performance during jump tasks. PLoS ONE. 2017;12(10): e0186297. 29020108 10.1371/journal.pone.0186297 PMC5636165

[59] Maloney SJ, Richards J, Fletcher IM. A comparison of bilateral and unilateral drop jumping tasks in the assessment of vertical stiffness. J Appl Biomech. 2018;34(3):199–204. 29364028 10.1123/jab.2017-0094

[60] Marey E-J. De la measure des forces dans les differents actes de la locomotion. Académie des Sci. 1883.

[61] Marey E-J, Demeny G. Locomotion humaine, mecanisme du saut. Acad. Sci. 1885.

[62] McHugh MP, Hickok M, Cohen JA, Virgile A, Connolly DAJ. Is there a biomechanically efficient vertical ground reaction force profile for countermovement jumps? Transl Sports Med. 2020;4(1):138–46.

[63] McMahon JJ, Suchomel TJ, Lake JP, Comfort P. Understanding the key phases of the countermovement jump force-time curve. Strength Cond J. 2018;40(4):96–106.

[64] McMahon JJ, Lake JP, Ripley NJ, Comfort P. Vertical jump testing in rugby league: a rationale for calculating take-off momentum. J Appl Biomech. 2020;36(6):370–4. 32796137 10.1123/jab.2020-0100

[65] McMahon JJ, Lake JP, Stratford C, Comfort P. A proposed method for evaluating drop jump performance with one force platform. Biomechanics. 2021;1(2):178–89.

[66] McMahon JJ, Lake JP, Comfort P. Identifying and reporting position-specific countermovement jump outcome and phase characteristics within rugby league. PLoS ONE. 2022;17(3): e0265999. 35333887 10.1371/journal.pone.0265999 PMC8956158

[67] Merrigan JJ, Stone JD, Hornsby WG, Hagen JA. Identifying reliable and relatable force-time metrics in athletes-considerations for the isometric mid-thigh pull and countermovement jump. Sports. 2020;9(1):4. 33396304 10.3390/sports9010004 PMC7824153

[68] Meylan CMP, Nosaka K, Green JP, Cronin JB. The effect of three different start thresholds on the kinematics and kinetics of a countermovement jump. J Strength Cond Res. 2011;25(4):1164–7. 20664368 10.1519/JSC.0b013e3181c699b9

[69] Moir GL. Three different methods of calculating vertical jump height from force platform data in men and women. Meas Phys Educ Exerc Sci. 2008;12(4):207–18.

[70] Moir GL, Gollie JM, Davis SE, Guers JJ, Witmer CA. The effects of load on system and lower-body joint kinetics during jump squats. Sports Biomech. 2012;11(4):492–506. 23259239 10.1080/14763141.2012.725426

[71] Montalvo S, Gonzalez MP, Dietze-Hermosa MS, Eggleston JD, Dorgo S. Common vertical jump and reactive strength index measuring devices: a validity and reliability analysis. J Strength Cond Res. 2021;35(5):1234–43. 33629975 10.1519/JSC.0000000000003988

[72] Morin J-B, Jiménez-Reyes P, Brughelli M, Samozino P. When jump height is not a good indicator of lower limb maximal power output: theoretical demonstration, experimental evidence and practical solutions. Sports Med. 2019;49:999–1006. 30805913 10.1007/s40279-019-01073-1

[73] Nakano N, Sakura T, Ueda K, Omura L, Kimura A, Iino Y, Fukashiro S, Yoshioka S. Evaluation of 3d markerless motion capture accuracy using openpose with multiple video cameras. Front Sports Act Liv. 2019;2:50. 10.3389/fspor.2020.00050 PMC7739760 33345042

[74] Niering M, Muehlbauer T. Differences in physical and psychological parameters in sub-elite, male, youth soccer players with jumper’s knee following physical therapy compared to healthy controls: A longitudinal examination. Int J Sports Phys Ther. 2021;16(1):114–25. 33604141 10.26603/001c.18658 PMC7872446

[75] Nikolaidou M-E, Marzilger R, Bohm S, Mersmann F, Arampatzis A. Operating length and velocity of human m. vastus lateralis fascicles during vertical jumping. R Soc Open Sci. 2017;4(5): 170185. 28573027 10.1098/rsos.170185 PMC5451828

[76] Oddsson L. What factors determine vertical jumping height? In: ISBS-conference proceedings archive. 1987.

[77] Pedley JS, Dicesare CA, Lloyd RS, Oliver JL, Ford KR, Hewett TE, Myer GD. Maturity alters drop vertical jump landing force-time profiles but not performance outcomes in adolescent females. Scand J Med Sci Sports. 2021;31(11):2055–63. 34275170 10.1111/sms.14025 PMC11148809

[78] Peng H-T, Song C-Y, Chen Z-R, Wang I-L, Gu C-Y, Wang L-I. Differences between bimodal and unimodal force-time curves during countermovement jump. Int J Sports Med. 2019;40(10):663–9. 31365944 10.1055/a-0970-9104

[79] Pérez-Castilla A, García-Ramos A. Evaluation of the most reliable procedure of determining jump height during the loaded countermovement jump exercise: take-off velocity vs. flight time. J Strength Cond Res. 2018;32(7):2025–30. 29570575 10.1519/JSC.0000000000002583

[80] Pérez-Castilla A, McMahon JJ, Comfort P, García-Ramos A. Assessment of loaded squat jump height with a free-weight barbell and smith machine: comparison of the take-off velocity and flight time procedures. J Strength Cond Res. 2017;34(3):671–7. 10.1519/JSC.0000000000002166 28777251

[81] Pérez-Castilla A, Rojas FJ, García-Ramos A. Reliability and magnitude of loaded countermovement jump performance variables: a technical examination of the jump threshold initiation. Sports Biomech. 2019;21(5):622–36. 31711369 10.1080/14763141.2019.1682649

[82] Pérez-Castilla A, Weakley JJS, García-Pinillos F, Rojas FJ, García-Ramos A. Influence of countermovement depth on the countermovement jump-derived reactive strength index modified. Eur J Sport Sci. 2020;21(12):1606–16. 33131460 10.1080/17461391.2020.1845815

[83] Pérez-Castilla A, Fernandes JFT, Rojas FJ, García-Ramos A. Reliability and magnitude of countermovement jump performance variables: influence of the take-off threshold. Meas Phys Educ Exerc Sci. 2021;25(3):227–35.

[84] Petridis L, Utczás K, Tróznai Z, Kalabiska I, Pálinkás G, Szabó T. Vertical jump performance in Hungarian male elite junior soccer players. Res Q Exerc Sport. 2019;90(2):251–7. 30901527 10.1080/02701367.2019.1588934

[85] Philpott LK, Forrester SE, van Lopik KA, Hayward S, Conway PP, West AA. Countermovement jump performance in elite male and female sprinters and high jumpers. Proc Inst Mech Eng Part P J Sports Eng Technol. 2021;235(2):131–8.

[86] Pinto BL, Callaghan JP. An appropriate criterion reveals that low pass filtering can improve the estimation of counter-movement jump height from force plate data. Meas Phys Educ Exerc Sci. 2021;25(4):344–52.

[87] Rago V, Brito J, Figueiredo P, Carvalho T, Fernandes T, Fonseca P, Rebelo A. Countermovement jump analysis using different portable devices: implications for field testing. Sports. 2018;6(3):91. 30200384 10.3390/sports6030091 PMC6162675

[88] Rojas AP-CFJ, García-Ramos A. Assessment of unloaded and loaded squat jump performance with a force platform: which jump starting threshold provides more reliable outcomes? J Biomech. 2019;92:19–28. 31126593 10.1016/j.jbiomech.2019.05.022

[89] Sargent DA. The physical test of a man. Am Phys Educ Rev. 1921;26:188–94.

[90] Street G, McMillan S, Board W, Rasmussen M, Heneghan JM. Sources of error in determining countermovement jump height with the impulse method. J Appl Biomech. 2001;17(1):43–54.

[91] Tenelsen F, Brueckner D, Muehlbauer T, Hagen M. Validity and reliability of an electronic contact mat for drop jump assessment in physically active adults. Sports. 2019;7(5):114. 31100833 10.3390/sports7050114 PMC6572231

[92] Van der Kruk E, Reijne MM. Accuracy of human motion capture systems for sport applications; state-of-the-art review. Eur J Sport Sci. 2018;18(6):806–19. 29741985 10.1080/17461391.2018.1463397

[93] Vanezis A, Lees A. A biomechanical analysis of good and poor performers of the vertical jump. Ergonomics. 2005;48(11–14):1594–603. 16338725 10.1080/00140130500101262

[94] Vanrenterghem J, Clercq DD, Cleven PV. Necessary precautions in measuring correct vertical jumping height by means of force plate measurements. Ergonomics. 2001;44(8):814–8. 11450878 10.1080/00140130118100

[95] Wade L, Lichtwark GA, Farris DJ. Movement strategies for countermovement jumping are potentially influenced by elastic energy stored and released from tendons. Sci Rep. 2018;8(1):2300. 29396499 10.1038/s41598-018-20387-0 PMC5797114

[96] Wade L, Lichtwark GA, Farris DJ. The influence of added mass on muscle activation and contractile mechanics during submaximal and maximal countermovement jumping in humans. J Exp Biol. 2019;222(2): jeb194852. 30651318 10.1242/jeb.194852

[97] Wade L, Lichtwark GA, Farris DJ. Comparisons of laboratory-based methods to calculate jump height and improvements to the field-based flight-time method. Scand J Med Sci Sports. 2020;30(1):31–7. 31544260 10.1111/sms.13556

[98] Wade L, Needham L, Mcguigan MP, Bilzon JLJ. Backward double integration is a valid method to calculate maximal and sub-maximal jump height. J Sports Sci. 2022;40(10):1191–7. 35356858 10.1080/02640414.2022.2059319

[99] Wank V, Coenning C. On the estimation of centre of gravity height in vertical jumping. German J Exerc Sport Res. 2019;49(4):454–62.

[100] Wilder JN, Riggins ER, Noble RA, Lelito CM, Widenhoefer TL, Almonroeder TG. The effects of drop vertical jump technique on landing and jumping kinetics and jump performance. J Electromyogr Kinesiol. 2021;56: 102504. 33242751 10.1016/j.jelekin.2020.102504

[101] Wisløff U, Castagna C, Helgerud J, Jones RL, Hoff J. Strong correlation of maximal squat strength with sprint performance and vertical jump height in elite soccer players. Br J Sports Med. 2004;38(3):285–8. 15155427 10.1136/bjsm.2002.002071 PMC1724821

[102] Xu J, Turner A, Comfort P, Harry JR, McMahon JJ, Chavda S, Bishop C. A systematic review of the different calculation methods for measuring jump height during the countermovement and drop jump tests. Sports Med. 2023;53(5):1055–72. 36940054 10.1007/s40279-023-01828-x PMC10115716

[103] Yamashita D, Murata M, Inaba Y. Effect of landing posture on jump height calculated from flight time. Appl Sci. 2020;10(3):776.

[104] Žiga Kozinc PJ. Discrepancy among different methods for vertical jump height determination and its implications for field-based testing: A narrative review. Meas Phys Educ Exerc Sci. 2022;27(3):248–56.

